# Effects of Different Maturation Systems on Bovine Oocyte Quality, Plasma Membrane Phospholipid Composition and Resistance to Vitrification and Warming

**DOI:** 10.1371/journal.pone.0130164

**Published:** 2015-06-24

**Authors:** José F. W. Sprícigo, Mateus N. Diógenes, Ligiane O. Leme, Ana L. Guimarães, Carolle V. Muterlle, Bianca Damiani Marques Silva, David Solà-Oriol, Ivo Pivato, Luciano Paulino Silva, Margot A. N. Dode

**Affiliations:** 1 School of Agriculture and Veterinary Medicine, University of Brasilia, Brasília-DF, Brazil; 2 Embrapa Genetic Resources and Biotechnology, Laboratory of Animal Reproduction, Brasília- DF, Brazil; 3 Servei de Nutrició i Benestar Animal (SNiBA), Departament de Ciència Animal i dels Aliments, Universitat Autònoma de Barcelona, 08193, Bellaterra, Spain; Inner Mongolia University, CHINA

## Abstract

The objective of this study was to evaluate the effects of different maturation systems on oocyte resistance after vitrification and on the phospholipid profile of the oocyte plasma membrane (PM). Four different maturation systems were tested: 1) in vitro maturation using immature oocytes aspirated from slaughterhouse ovaries (CONT; n = 136); 2) in vitro maturation using immature oocytes obtained by ovum pick-up (OPU) from unstimulated heifers (IMA; n = 433); 3) in vitro maturation using immature oocytes obtained by OPU from stimulated heifers (FSH; n = 444); and 4) in vivo maturation using oocytes obtained from heifers stimulated 24 hours prior by an injection of GnRH (MII; n = 658). A sample of matured oocytes from each fresh group was analyzed by matrix associated laser desorption-ionization (MALDI-TOF) to determine their PM composition. Then, half of the matured oocytes from each group were vitrified/warmed (CONT VIT, IMA VIT, FSH VIT and MII VIT), while the other half were used as fresh controls. Afterwards, the eight groups underwent IVF and IVC, and blastocyst development was assessed at D2, D7 and D8. A chi-square test was used to compare embryo development between the groups. Corresponding phospholipid ion intensity was expressed in arbitrary units, and following principal components analyses (PCA) the data were distributed on a 3D graph. Oocytes obtained from superstimulated animals showed a greater rate of developmental (P<0.05) at D7 (MII = 62.4±17.5% and FSH = 58.8±16.1%) compared to those obtained from unstimulated animals (CONT = 37.9±8.5% and IMA = 50.6±14.4%). However, the maturation system did not affect the resistance of oocytes to vitrification because the blastocyst rate at D7 was similar (P>0.05) for all groups (CONT VIT = 2.8±3.5%, IMA VIT = 2.9±4.0%, FSH VIT = 4.3±7.2% and MII VIT = 3.6±7.2%). MALDI-TOF revealed that oocytes from all maturation groups had similar phospholipid contents, except for 760.6 ([PC (34:1) + H]^+^), which was more highly expressed in MII compared to FSH (P<0.05). The results suggest that although maturation systems improve embryonic development, they do not change the PM composition nor the resistance of bovine oocytes to vitrification.

## Introduction

The ability to preserve female gametes is an integral part of assisted reproductive techniques (ARTs) and can have a significant impact on animal conservation programs, animal breeding programs, and human-assisted conception [1, 2].

For animal production, oocyte cryopreservation is crucial for overcoming the logistical problems associated with the numbers of recovered oocytes, their transportation to the lab and the availability of recipients for the produced embryos. These issues are of particular concern in large-scale commercial embryo production programs. In addition, oocyte cryopreservation allows for the storage of unfertilized genetic material from a female until her potential can be evaluated, allowing for commercialization while avoiding animal transportation and sanitary risks.

To date, oocyte cryopreservation remains an ineffective technique for most domestic animals, due to the very low ability of cryopreserved oocytes from most species to undergo proper embryonic development. This high sensitivity of oocytes to cryopreservation could be explained by their unique morphological characteristics such as cell size, cytoplasmic water volume and cytoskeletal organization [3–10]. Therefore, cryopreserved oocytes may suffer severe morphological and functional damage that can be exacerbated due to the high cytoplasmic lipid content and to the phospholipid composition of the membrane [11].

Currently, vitrification is the most widely used technique for preserving oocytes. While it has already been established for human oocytes with remarkable results [12], the results are still very poor for bovine oocytes [2, 13, 14]. When comparing human and bovine oocytes, the most obvious differences are the oocyte maturation system, which is usually in vivo for humans [13] and in vitro for bovines; the amount of cytoplasmic lipids [26], which are much greater in bovines than in humans; and the composition of the lipid plasma membrane.

Therefore to approximate the vitrification results obtained with human oocytes, those characteristics should be the focus when studying bovine oocytes. Over the last decade, many efforts have been made to increase the vitrification efficiency for farm animal oocytes [1, 8] including the use of lipolytic agents [15, 16] and plasma membrane (PM) modifiers [4, 14]. The reduction of cytoplasmic lipid droplets can decrease the damage caused by cryopreservation to organelles such as the mitochondria, endoplasmic reticulum, endosomes, peroxisomes, and the cytoskeleton [17]. Modifications of the PM may affect cell permeability, improving the exchange of water and cryoprotectors [4]. Phospholipids, particularly phosphatidylcholines (PC) and sphingomyelins (SM), are structural units of the functional PM, and their composition determines most physico-chemical properties of the cell membrane including fluidity, permeability and thermal phase behavior [18]. However, the use of oocytes matured in vivo, which would be expected to be of higher quality, in vitrification has not yet been reported in bovines.

Moreover, the fatty acid (FA) profile of cattle and pig oocytes have been shown to change based on culture media composition, and these different composition are related to oocyte developmental competence [19, 20]. Sudano *et al*. [21] have shown that the origin of embryos, produced either in vivo or in vitro, affects lipid composition. However, it is not known whether the same changes occur in the PM of bovine oocytes.

The main objective of this study was to compare the resistance of oocytes that had been maturated using different systems to vitrification and warming by evaluating blastocyst development and quality after in vitro fertilization (IVF) and culture (IVC). In addition, we focused on the effects of the different maturation systems on the follicular population, size and vascularity, prior to oocyte pick-up (OPU) and also oocyte quality and compared the PL present in the plasma membrane of the retrieved oocytes. We used the vivo maturation system as the standard, which results in oocytes with greater capacity for embryonic development [22–24] and consequently more resistance to vitrification.

## Materials and Methods

All of the experiments, including the pre-experiment, were conducted according to Brazilian laws for animal ethics and health research and approved by the Institutional Animal Care and Use Committee (IACUC) of the Institute of Biological Sciences, University of Brasilia under UnBDOC protocol (process number 132157/2012).

Unless otherwise specified, all reagents were purchased from Sigma-Aldrich (St. Louis, MO, USA). Vitrification devices were acquired from Ingámed (Maringá, PR, Brazil). The IVP media, including media for in vitro maturation, in vitro fertilization and in vitro culture were produced by Geneal (Geneal, Animal Genetics, Uberaba-MG, Brazil).

### Experimental design

Before initiating the experiment, a pre-experiment was conducted to determine the best interval to perform ovum pick-up (OPU) for the oocytes matured in vivo. Briefly, Nellore heifers were synchronized, superstimulated and given the GnRH administration as described below in the in vivo maturation protocol. Following GnRH administration at 20 (n = 5) or 24 (n = 5) hours, the animals were subjected to OPU, and the retrieved COCs were denuded and evaluated for the presence of a polar body. Next, they were fixed and stained to determine their meiotic stage. The group that showed a greater percentage of oocytes at the MII stage was chosen for the experiment.

A total of 1,562 oocytes were used to assess whether the maturation system affects oocyte viability and membrane phospholipid composition after vitrification/warming. Three maturation systems were evaluated: 1) in vitro maturation of oocytes obtained from unstimulated heifers by OPU (IMA); 2) in vitro maturation of oocytes obtained from stimulated heifers by OPU, in which OPU was performed 12 hours after the last FSH administration (FSH); 3) in vivo maturation in which oocytes were obtained from stimulated heifers, with OPU performed 24 hours after GnRH administration (MII). A fourth group was also used as a laboratory control group (CONT), consisting of the in vitro maturation of oocytes aspirated from slaughterhouse ovaries.

The experiment was conducted with nine replicates in each of which five animals from each of the three groups were subjected to OPU in such a way that animals from all groups were present in the replicate. For the IMA and FSH groups (immature oocytes), the COCs were retrieved, selected, transferred to the IVM medium and matured for 24 hours. For the in vivo matured group (MII), heifers were subjected to OPU, and mature oocytes were recovered 24 hours later than for the other two groups.

Just before each OPU section, both ovaries from the animals from groups IMA, FSH and MII were evaluated using a color Doppler ultrasound (30 MyLab Vet Gold, Esaote, Italy). The ovaries were scanned, and the follicles were counted and graded according to the intensity of blood vascularization.

The oocytes from all of the treatments were classified as either viable or non-viable. For oocytes from the IMA and FSH groups, only those with more than three cumulus cell layers and homogeneous cytoplasm were classified as viable. For the MII group, only oocytes with homogeneous cytoplasm, a presentation of the first polar body extrusion and/or cumulus cell expansion were considered viable. Among the non-viable oocytes, those presenting signals of vacuolization were classified as degenerated.

**Fig 1:**
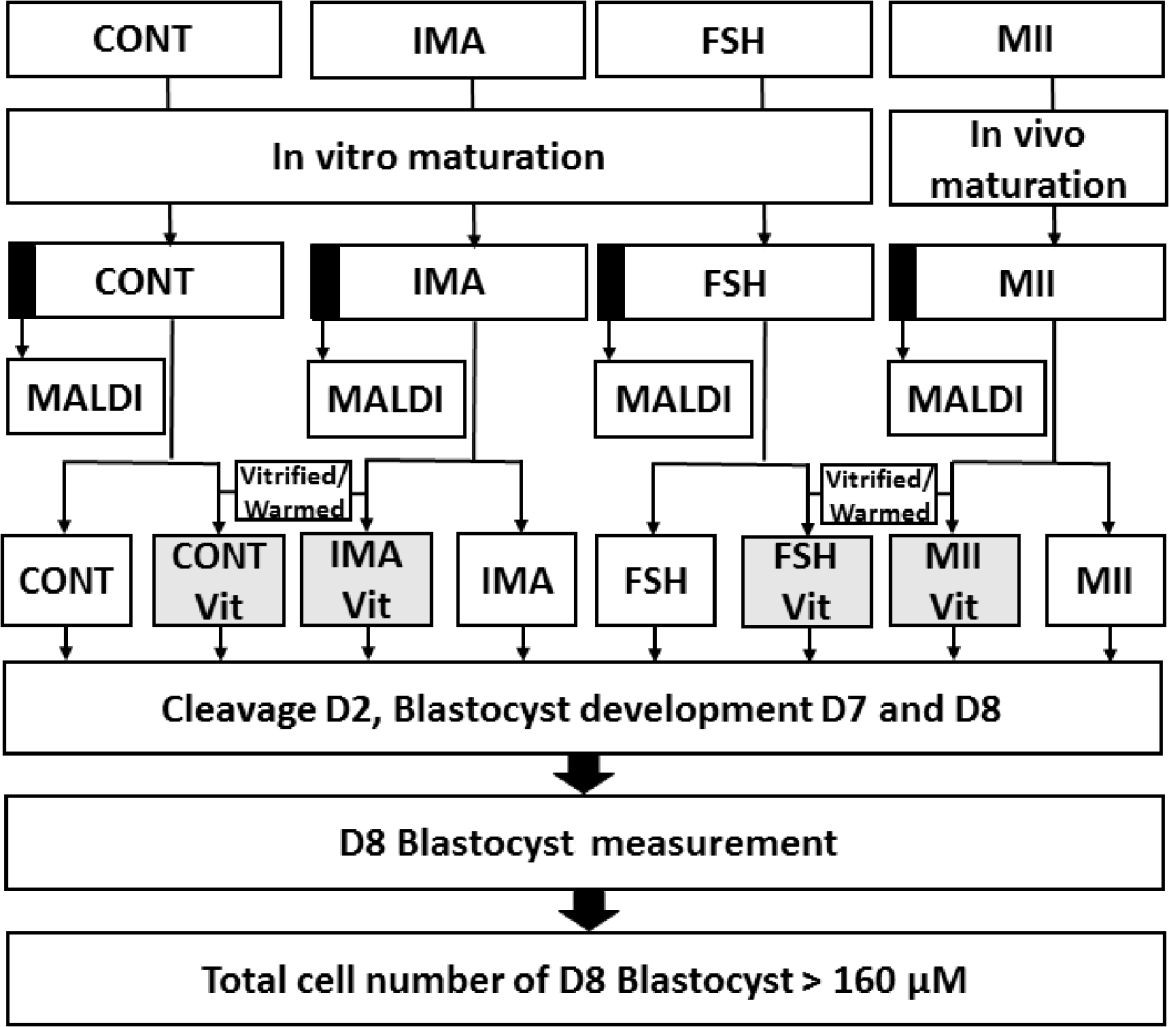
Experimental design flow diagram. Flow diagram of experimental design for the different treatments. Oocytes recovered from slaughterhouse ovaries (CONT), obtained by OPU from non-superstimulated females (IMA) and from superstimulated females (FSH) were matured in vitro. In vivo-matured oocytes were obtained by OPU from superstimulated females that received an ovulation inducer 24 hours previously (MII). A sample of matured oocytes from each of four groups was used to study the composition of plasma membrane phospholipids using MALDI-TOF. The remaining oocytes were divided in half, one half consisting of non-vitrified fresh oocytes (CONT, IMA, FSH and MII) and other of vitrified/ warmed oocytes (CONT Vit, IMA Vit, FSH Vit and MII Vit). At the end of the warming process, the eight groups were used for in vitro fertilization and culture. Cleavage at D2 and blastocyst development at D7 and D8 were evaluated. At D8, all of the blastocysts were measured, and those larger than 160 μM in diameter were stained for total cell number counting.

Fig 1 represents the experimental design. Following in vivo or in vitro maturation, half of the oocytes from each treatment were vitrified (VIT) and immediately warmed (CONT-VIT,-IMA-VIT,-FSH-VIT and MII-VIT). The other half was kept on the bench during the vitrification/warming process. For consistency during the entire experiment, only COCs with a visible polar body and/or cumulus cell expansion were classified as in vivo-matured and were used in the MII group. After warming, cryopreserved oocytes and their fresh controls underwent in vitro fertilization and embryo culture. The cleavage rate was evaluated 48 hours after fertilization (D2), and embryonic development was assessed at D7 and D8. At D8, embryos were measured and stained to count the total cell number.

Samples of fresh oocytes (n = 15/treatment) from each group (CONT, IMA, FSH and MII) were collected for phospholipid evaluation using mass spectrometry fingerprint profiles (MALDI / TOF).

### Oocyte recovery from slaughterhouse ovaries

Ovaries (*Bos indicus*) were collected immediately after slaughter (Qualimaxima, Brasilia-DF, Brazil) and transported to the laboratory in saline solution (0.9% NaCl) supplemented with penicillin G (100 IU/mL) and streptomycin sulfate (100 g/mL) at 35°C. Cumulus oocyte complexes (COCs) were aspirated from 3- to 8-mm diameter follicles with an 18-gauge needle and pooled in a 15-mL conical tube. COCs were recovered and selected in holding medium consisting of HEPES-buffered TCM-199 (Gibco BRL, Burlington, ON, Canada) supplemented with 10% fetal calf serum [FCS (Invitrogen, Carlsbad, CA, USA)]. Only COCs with homogenous cytoplasm and at least three layers of cumulus cells were used for the experiments. After selection, oocytes were subjected to in vitro maturation.

### Oocyte recovery by OPU

A total of 43 Nellore heifers (*Bos taurus indicus*) that were approximately 2 years old and had similar body conditions were used. The heifers were maintained on *Brachiaria sp.* pasture and supplemented with corn silage, salt and water *ad libitum*. Heifers were randomly assigned into three experimental groups (n = 14; n = 14 and n = 15) in a crossover design with a minimal interval between replicates of 30 days.

For OPU, we used an ultrasound device (Aloka SSD 500, Japan) coupled to a micro convex sector transducer at 7.5 MHz (Aloka, UST 9125, Japan). The recovered COCs were selected under a stereomicroscope (Zeiss—Stemi SV6, Germany) and subjected to either IVM or vitrification treatment.

### In vitro maturation (IVM)

Selected COCs were washed and transferred in batches of 25–30 complexes to a 200-μL drop of maturation medium under silicone oil and incubated for 22 h at 39°C with 5% CO_2_ in air. The maturation medium consisted of TCM –199 (Invitrogen, Carlsbad, CA, USA) supplemented with 10% fetal bovine serum (FBS), 0.01 IU/mL of FSH, 0.1 mg/mL of L-glutamine and antibiotics (amicacyn, 0.075 mg/mL).

### Estrus synchronization, ovarian stimulation and in vivo maturation

After establishing the appropriate time regardless of treatment for the retrieval of in vivo-matured oocytes [23, 25], all animals were subjected to identical protocols for estrus synchronization. The animals received an i.m. injection of 2 mg of estradiol benzoate (Ric-BE; Syntex, Buenos Aires, Argentina) and an intravaginal progesterone-releasing device (Sincrogest; Ourofino, Cravinhos-SP, Brazil) was inserted. This was considered Day -10 of the protocol. Five days later (D-5), the animals received an injection of 0.150 mg of prostaglandin PGF2α (d-cloprostenol, Prolise ARSA SRL, Argentina). On D-2, the progesterone implants were removed, and on day -1, we injected1 mg of estradiol benzoate (i.m.). D0 was considered the beginning of the treatment.

On Day 8, all follicles larger than 5 mm in diameter were removed by trans-vaginal aspiration. Immediately after ablation, the animals received an intravaginal progesterone-releasing device. For the non-stimulated animals (IMA group), 108 hours after the progesterone device was inserted, we administered 0.150 mg of PGF2α (i.m.). After 72 h, the progesterone device was removed, and OPU was performed. All follicles larger than 3 mm in the ovaries were aspirated.

For the other two groups, animals were superstimulated two days after ablation (D10) by a total of 80 mg of pFSH (Folltropin-V; Bioniche Animal Health Canada Inc., Belleville, Canada) given as twice daily injections over four days in a decreasing dose schedule. Luteolysis was induced with 0.150 mg of PGF2α (i.m.) injected at the time of the fifth injection of pFSH. During the final injection, the progesterone device was removed, and OPU was performed.

To obtain in vivo-matured oocytes (MII group), 0.025 mg (i.m.) of lecirelin (Gestran, Tecnopec, São Paulo, Brazil), a GnRH analogue, was injected simultaneously with the final injection of FSH and the removal of the progesterone device. OPU was performed 24 hours later, and the follicles larger than 6 mm were aspirated.

### Evaluation of follicular blood vascularity

In all treatments, the ovaries were evaluated just prior to the OPU procedure. A color Doppler ultrasound with a 7.5-MHz linear probe was used for the evaluation. Vascularization was observed and classified according to Matsui *et al*. [26] as follows: 3- vascularization was absent, and no colored images surrounding the follicle were observed; 2- intermediate vascularization, with partially colored images; and 1- intense vascularization, when the follicle was completely surrounded by colored images.

### In vitro fertilization (IVF) and embryo culture (IVC)

Following maturation, COCs (in groups of 25 to 30) were transferred to a 200-μL drop of fertilization medium. Frozen semen from a Nellore bull previously tested in our laboratory for IVF was used for fertilization. Motile spermatozoa were obtained by the Percoll method [27] and were added to the droplets containing COCs at a final concentration of 1×10^6^ spermatozoa mL^−1^. The fertilization medium was TALP [28] supplemented with penicillamine (2 mM), hypotaurine (1 mM), epinephrine (250 mM) and heparin (10 μg/mL). Spermatozoa and oocytes were co-incubated for 18 h at 39°C with 5% CO_2_ in air. The day of *in vitro* insemination was considered to be day 0. After co-incubation, the presumptive zygotes (n = 25–30) were washed, transferred to 200-μL drops of SOFaaci medium [29] supplemented with 2.77 mM of myo-inositol and 5% FBS and cultured at 39°C and 5% CO_2_ in the air for 8 days. Blastocyst development was evaluated on Day 2 post-insemination (pi) for cleavage and at Day 7 and Day 8 for the blastocyst rate.

### Vitrification and warming

Oocyte vitrification was performed as previously described [1] with slight modifications. The holding medium (HM), which was used to handle oocytes during vitrification and warming, was composed of HEPES-buffered TCM-199 (Gibco) supplemented with 20% FCS. For vitrification, the groups were first washed in an equilibrium solution composed of 7.5% ethylene glycol and 7.5% dimethylsulfoxide (DMSO). Oocytes were transferred to a vitrification solution consisting of 15% ethylene glycol, 15% DMSO and 0.5 M sucrose in HM. Next, the oocytes were placed into a cryotop device in sets of 3–5 under a stereomicroscope (Nikon- SMZ 650), and the device was immediately submerged into liquid nitrogen. Warming was performed immediately after vitrification by immersing the cryotop end for 1 min into a drop of HM that was pre-warmed to 37 °C and supplemented with 1 M sucrose. The oocytes were transferred to HM medium supplemented with 0.5 M sucrose for 3 min and finally to the original holding medium. Afterwards, the oocytes were placed into culture dishes for IVF.

### Assessment of maturation

For the assessment of maturation, COCs punctured 20 or 24 hours after the administration of GnRH were denuded and fixed for at least 48 h with acetic alcohol (1:3). On the day of the evaluation, the oocytes were placed on a slide, covered with a coverslip and stained with 1% lacmoid in 45% glacial acetic acid. The maturation stage of each oocyte was determined using phase contrast microscopy. Oocytes were classified as follows: immature—did not reach metaphase II or mature–having reached metaphase II.

### Embryo measurement and cell number

After IVF, presumptive zygotes were cultured to D8, when the blastocysts were classified and measured by a motic image camera Motic-Moticam 2.0 Plus, Japan. Blastocysts with a diameter >160 μm were used to evaluate the total number of cells by exposure to Hoechst 33342 dye (Invitrogen, Carlsbad, CA, USA) at a concentration of 1 μg/mL for 5 min. Subsequently the blastocysts were transferred to a slide and covered with a cover slip. The slides were evaluated using an epifluorescence microscope (Zeiss Axiophot, Germany, filter 24) with a wavelength of 494/518 nm (excitation/emission), and the cell nuclei were counted.

### Lipid mass spectrometry MALDI-TOF

#### Sample preparation

Matured oocytes from the different treatments were completely stripped of cumulus cells after 3 minutes in a solution of 1% hyaluronidase. Each oocyte was washed five times in drops of 1:1 methanol and pure water (v/v) and stored in the same solution in micro centrifuge tubes at -80°C until analysis. After thawing, the oocytes were allocated individually into a MALDI target well of a 96-well steel plate, and the oocytes were allowed to dry at room temperature. Prior to analysis, 1 μl of 2,5-dihydroxybenzoic acid (0.125 M, DHB) diluted in pure methanol was deposited into each well to cover the oocytes and to allow for crystallization, and the oocytes were again allowed to dry at room temperature.

#### MALDI-TOF

The spectra obtained with MALDI-TOF mass spectrometry were acquired in positive mode by a reflected Auto Flex Speed MALDI-TOF/TOF mass spectrometer (Bruker Daltonics, Bremen, Germany). Data were acquired in a mass range from 700–900 m/z with 1500 laser shots in different oocyte regions. The laser was applied until all signs had disappeared in the region of interest due to sample desorption. The laser intensity was standardized at 40% for the spectrum acquisition in all the samples. Spectra were centered and aligned using mMass 5.5.0 software [30]. The most intense ions upon the detection of peaks corresponding to isotopic distributions were considered as the starting point corresponding to the lipid ions. For the experiments, a total of 10–15 oocytes per group of fresh oocytes from the four different systems were used; no vitrified and warmed oocytes were evaluated.

#### Determination of plasma membrane phospholipids

The MALDI-TOF analyses identified the ionic intensity after biological material ionization, and each peak of ions corresponded to one different molecule. The experiment was conducted to investigate the compounds present between 700 and 900 *m/z*. Within this interval, phospholipids, primarily phosphatidylcholines and sphingomyelins (PC and SM), are frequently observed [21]. To determine which phospholipid corresponded to each peak, the *m/z* values obtained by MALDI-TOF were compared to values reported recently in the literature [21, 31–33] and to those of an online database (www.lipidmaps.org).

### Statistical analyses

All data were analyzed using Statistical Analysis System software (SAS, 1999). Data for the evaluation of the oocyte maturational status during the pre-experiment were analyzed by the Chi-square test with a 5% significance level. Data from follicular population, size and vascularization, blastocyst diameter and total cell number did not present a normal distribution and were compared using the Kruskall-Wallis test with a 5% significance level. For embryo development at D2, D7, and D8 and for hatching rates at D8, the variables were analyzed using the GENMOD procedure using the statistical package SAS v.9.2. The model used included the experimental treatments as the main effect. All pairwise differences were analyzed using LSMEANS based on a Chi-square inference as a test of comparisons with 5% significance level.

To analyze the MALDI-TOF data, a principal component analysis (PCA) was first performed using the PRINCOMP procedure. Loadings and scores were plotted on a 3D graph, and the most disperse ion intensities and their relative means were compared using an ANOVA taking into account Tukey’s adjustment for the LSMEANS and a 5% significance.

## Results

### Determination of the ideal moment for in vivo MII oocyte acquisition

After lacmoid staining, a greater percentage (P <0.05) of matured oocytes (MII) was observed when the follicles were aspirated at 24 hours (85%; n = 31) following the administration of GnRH compared to those aspirated after 20 hours (31%; n = 34).

#### Follicular number and blood vascularization for the different treatments at the time of OPU and oocyte recovery

Initially, we compared the availability of follicles to be aspirated in the ovaries at the time of OPU between the treatments. As shown in Table 1, we observed no difference in the average number of follicles present in the ovaries. However, when the follicular diameter was considered, the IMA group showed the greatest number of follicles that were < 6 mm (P <0.05). The population of follicle that were > 6 mm in diameter was similar between the FSH and MII groups and smallest in the IMA group (P< 0.05).

**Table 1:** Total number (N), mean and standard deviation (±SD) of follicles per female of different diameters aspirated by ovum pick-up, after nine replicates, from the ovaries of non-superstimulated (IMA), superstimulated (FSH) and superstimulated females that received an ovulation inducer (MII).

			Follicles Diameter	
Groups	Number of follicles		<6 mm	>6mm
	N	Means (±SD)	Means (±SD)	Means (±SD)
IMA	852	20.2 (±11.5) ^a^	19.7 (±11.4)^a^	0.6 (±0.8)^b^
FSH	914	22.8 (±11.7) ^a^	2.1 (±3.5)^b^	20.7 (±12.1)^a^
MII	1013	22.5 (±15.0)^a^	-	22.5 (±15.1)^a^

^a,b^ Values with different superscripts in the same column are significantly different by Kruskall-Wallis (P < 0.05).

We also evaluated the level of follicle vascularization in all treatment groups prior to OPU. In the group of animals that had not received ovarian superstimulation (IMA), the majority of the follicles showed an absence of vascularization (P <0.05), and the mean number of follicles with intense blood flow was lower (P <0.05) than in the other groups. The group in which animals received only FSH had the greatest number (P <0.05) of follicles with moderate vascularization, while those that received FSH and GnRH (MII group) had the greatest (P <0.05) number of follicles with intense vascularization (Table 2).

**Table 2:** Total number (N), mean and standard deviation (±SD) of follicles per female evaluated and classified by the color Doppler, after nine replicates, as having intense, moderate or absent blood vascularization in the ovaries of non-superstimulated (IMA), superstimulated (FSH) and superstimulated females that received an ovulation inducer (MII).

			Blood follicular vascularization		
Groups	Number of follicles		Intense	Moderate	Absent
	N	Means (±SD)	Means (±SD)	Means (±SD)	Means (±SD)
IMA	1016	24.1(±10.0) ^a^	0.3 (±0.8)^c^	6.7 (±3.6)^c^	17.1 (±7.7)^a^
FSH	1098	27.4(±12.4) ^a^	4.1 (±4.2)^b^	16.7 (±8.5)^a^	6.6(±4.8)^b^
MII	1341	29.8(±17.0) ^a^	16.7 (±12.2)^a^	11.9 (±8.7)^b^	1.1 (±1.8)^c^

^a,b,c^ Values with different superscripts in the same column are significantly different by Kruskall-Wallis test (P < 0.05).

With regard to the viability of recovered COCs (Table 3), the FSH group (n = 444) had a greater percentage (P <0.05) of viable COCs compared to the MII (n = 658) and IMA (n = 433) groups; there was no difference between the MII and IMA groups. However, when evaluating the non-viable oocytes, the MII group had the lowest (P<0.05) percentage of degenerated oocytes, while most of the non-viable oocytes (P<0.05) had degenerated in the other groups.

**Table 3:** Total number (N) and percentage (%) of viable and nonviable oocytes recovered by ovum pick-up, after nine replicates, from the ovaries of non-superstimulated (IMA), superstimulated (FSH) and superstimulated females that received an ovulation inducer (MII).

			Non-viable oocytes	
Groups	Total N	Viable N (%)	N (%)^*^	Degenerated/Non-viable** N (%)
IMA	433	235 (54.3)^b^	198 (45.7)^a^	155 (78.3)^b^
FSH	444	337 (75.9)^a^	107 (24.1)^c^	99 (92.5)^a^
MII	658	438 (66.6)^b^	220 (33.4)^b^	63 (28.6)^c^

^a,b,c^ Values with different superscripts in the same column are significantly different, by to Chi—square test (P < 0.05).

* Only those oocytes presenting less than three cumulus cell layers or heterogeneous cytoplasm were classified as non-viable for IMA and FSH groups. For the MII group, only oocytes with heterogeneous cytoplasm or without the first polar body extrusion and/or cumulus cell expansion were considered non-viable. The percentage is expressed as the ratio to the total number of recovered oocytes.

**Oocytes with heterogeneous cytoplasm and presenting vacuolization at the three experimental groups were classified as degenerated. The percentage is expressed as the ratio of the total number of non-viable oocytes.

### Embryo development after vitrification/warming and the phospholipid composition of oocytes from different maturation systems

The resistance of oocytes to vitrification/warming that were obtained using different maturation systems was evaluated by embryo development (Table 4). Although the cleavage rate was higher in the CONT-VIT group (P<0.05), embryo development at D7 and D8 was similar among all vitrified groups and lower (P<0.05) compared to all fresh groups. In contrast, the hatching rate at D8 for the FSH-VIT and MII-VIT groups was similar to the fresh CONT group. The cleavage rates were similar between all fresh oocyte groups and higher than the all of the vitrified groups (P<0.05). However, the maturation system had an effect on the proportion of D7 and D8 blastocysts obtained from fresh oocytes, with a higher blastocyst rate from the FSH and MII groups (P<0.05) compared to the CONT and IMA groups. Further analysis revealed that among the fresh oocytes, the CONT group had the lowest (P<0.05) hatching rate and the MII and FSH groups had the highest rates (P<0.05) at D8.

**Table 4:** Blastocyst development of oocytes recovered from slaughterhouse ovaries (CONT) and by ovum pick-up (OPU) from the ovaries of non-superstimulated females (IMA), superstimulated females (FSH) and superstimulated females that received an ovulation inducer (MII) that were vitrified (VIT) at the metaphase II stage.

		D2 Cleavage	D7 Blastocysts	D8 Blastocysts	D8* Hatched Blastocysts
Groups	Total	N	N	N	N
	N	(% ± SD)	(% ± SD)	(% ± SD)	(% ± SD)
CONT	136	110	52	52	13
		(79.4±10.7)^a^	(37.9±8.5)^b^	(37.9±8.5)^b^	(9.0±6.1)^c^
IMA	97	78	48	49	19
		(82.2±12.0)^a^	(50.6±14.4)^b^	(51.4±13.9)^b^	(19.6±13.8)^b^
FSH	128	111	73	75	24
		(86.3±10.3)^a^	(58.8±16.1) ^a^	(60±15.5)^a^	(20.7±14.9)^a^ ^b^
MII	212	176	132	133	77
		(83.6±14.8)^a^	(62.4±17.5)^a^	(62.7±17.2)^a^	(38.9±23.2)^a^
CONT VIT	101	25	3	4	2
		(25.7±5.6)^b^	(2.8±3.53)^c^	(3.6±4.8)^c^	(1.8±3.2)^d^
IMA VIT	92	15	3	3	3
		(16.4±7.2)^c^	(2.9±4.0)^c^	(2.9±4.0)^c^	(2.8±8.1)^d^
FSH VIT	111	18	4	4	1
		(14.5±8.92)^c^	(4.3±7.2)^c^	(4.3±7.2)^c^	(3.6± 2.6)^c^ ^d^
MII VIT	139	19	5	6	5
		(14.3±8.9)^c^	(3.6±7.2) ^c^	(4.5±7.2)^c^	(3.5± 2.1)^c^ ^d^

^a,b,c,d^ Values with different superscripts in the same column are different at P<0.05.

* Hatched blastocyst at D8 as a percentage of oocyte number.

On D8, all embryos were measured, and those with a diameter greater than 160 μm were analyzed for total cell number. Due to the low rates of embryo development in all vitrified groups, there was not sufficient number of embryos to perform a statistical analysis. The embryo quality among fresh oocytes, as evaluated by embryo size and total cell number, was similar for all the treatments. The only difference observed was that the MII embryos presented a higher cell number (P <0.05) than the CONT group (Table 5).

**Table 5:** Percentage, mean (μm) and standard deviation (SD) of size (μm) and total cell number of D8 blastocyst with diameters > 160 μm derived from oocytes of different maturation conditions: slaughterhouse ovaries (CONT) and by OPU, from ovaries of non-superstimulated females (IMA), superstimulated females (FSH) and superstimulated females that had received a ovulatory inducer (MII).

	Blastocysts	Blastocysts diameter	Blastocysts > 160μm	
Groups	N	μm (±SD)	N*	Total cell number
				mean (±SD)
CONT	42	188 (±36.6) ^a^	39	160 (±49.0)^b^
IMA	47	201 (±34.5)^a^	37	167 (±40.5)^a^ ^b^
FSH	67	197 (±32.3)^a^	61	192 (±45.6)^a^ ^b^
MII	113	211 (±40.2)^a^	100	194 (±24.8)^a^

^a,b,c,d^ Values with different superscripts in the same column are significantly different by Kruskall-Wallis test (P < 0.05).

CONT = oocytes from slaughterhouse ovaries that were matured in vitro; IMA = OPU oocytes from non-stimulated animals matured in vitro; FSH = OPU oocytes from FSH simulated animal and matured in vitro; MII = OPU oocytes after in vivo maturation.

*Represents to the quantity of embryos that was able to be evaluated in the counting of total cell numbers because some embryos were lost during staining or could not be observed.

**Fig 2:**
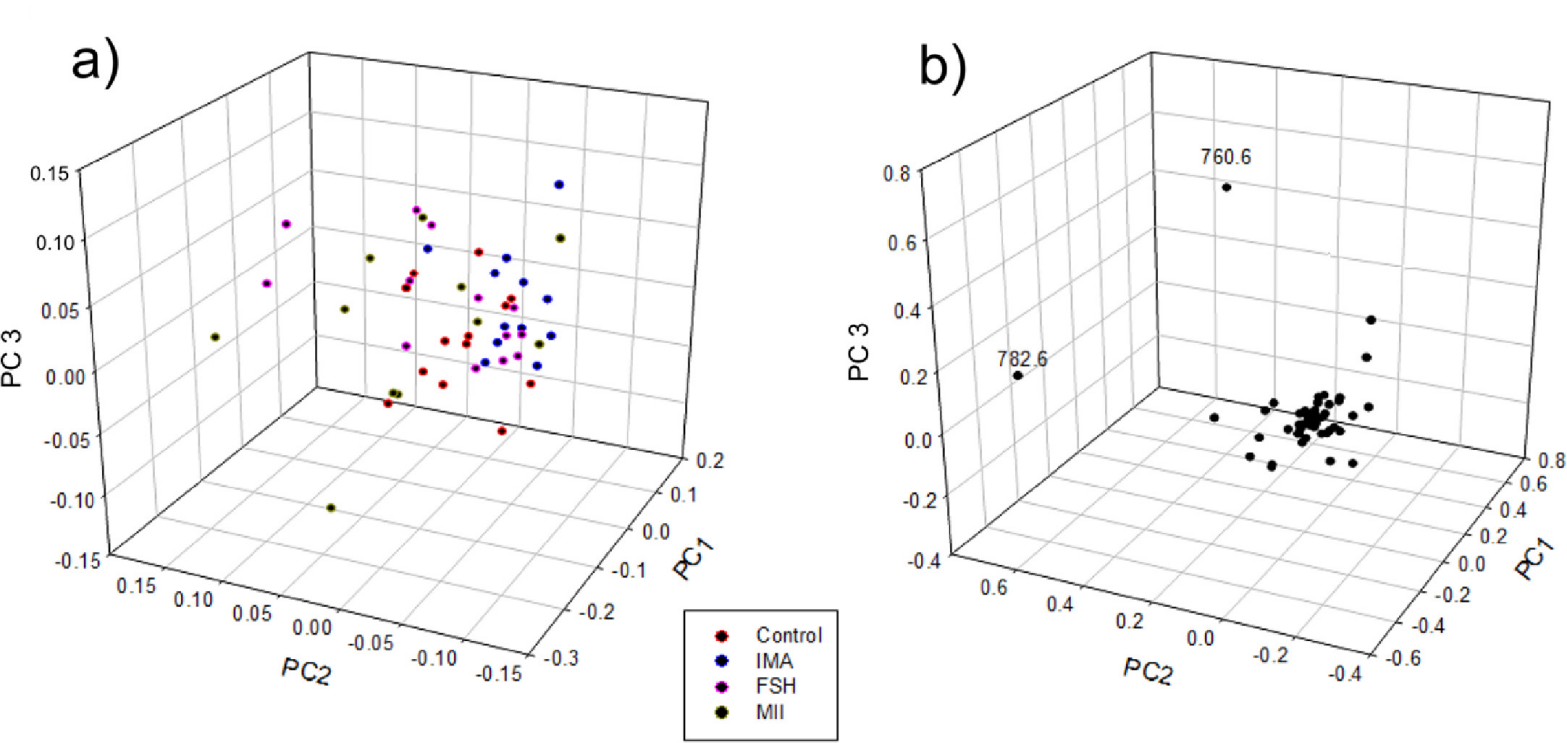
3D PCA plot for MALDI-TOF data of individual oocytes from different maturation systems. Shown is a 3D PCA plot for the MALDI-TOF data of single oocytes. a) Red (n = 12), blue (n = 13), pink (n = 13) and dark yellow (n = 10). Each point indicates the 3D PCA plot of an oocyte based on its phospholipid composition. The following four fresh oocyte experimental groups are represented: immature and in vitro-matured oocytes recovered from slaughter house ovaries (CONT), oocytes obtained by OPU from non-superstimulated females (IMA), superstimulated females (FSH) and in vivo-matured oocytes obtained by OPU from superstimulated females that received an ovulation inducer (MI). b) indicates the main ions represented, 760.6[PC (34:1) + H]^+^ and 782.6 [PC (34:6) + H]^+^ or [PC (34:1) + Na]^+^, are responsible for the most variability between the treatments. The three principal components explain >73% of the variability of the data.

**Fig 3:**
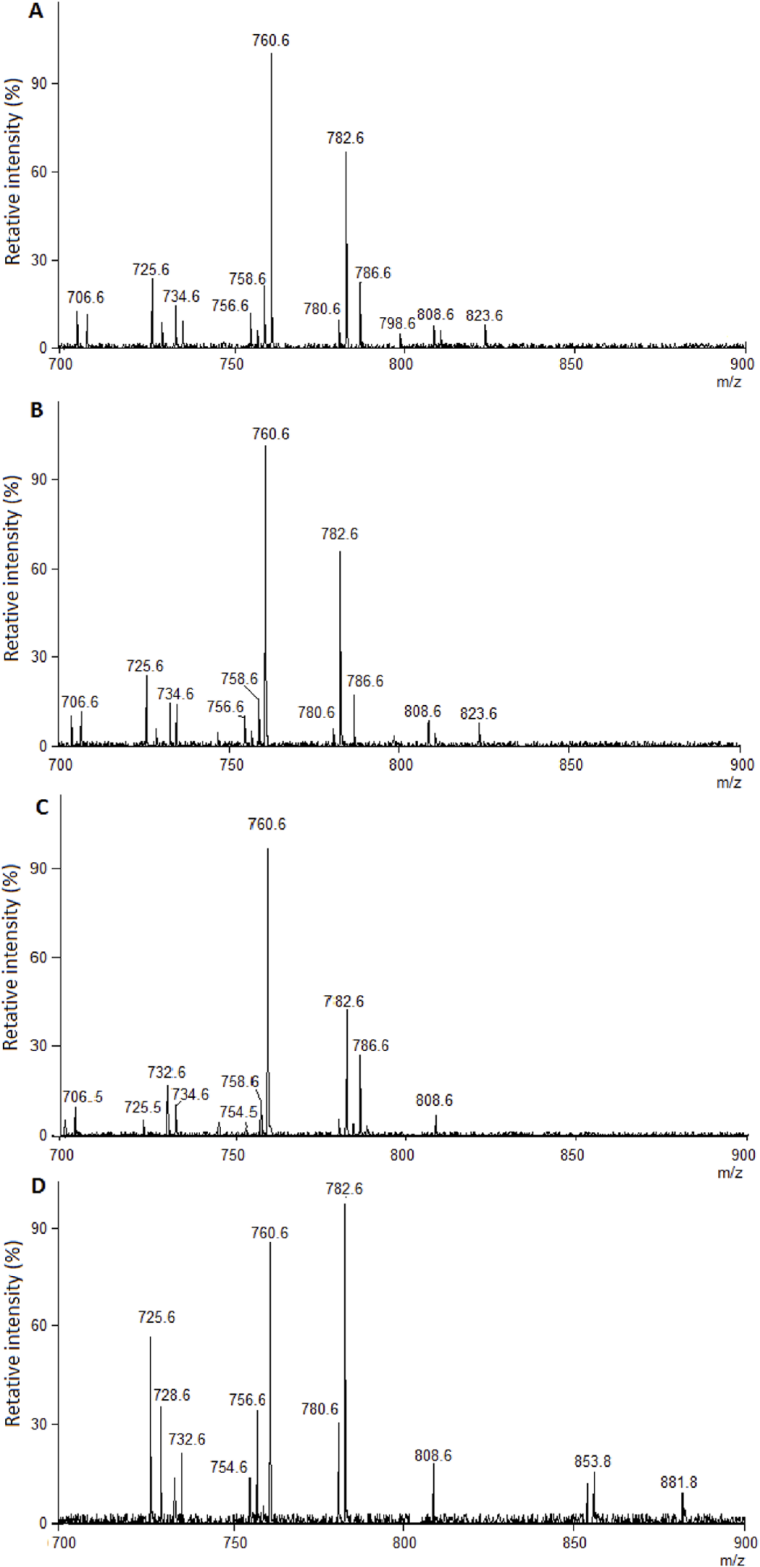
Representation of MALDI-TOF spectra of bovine oocytes from different systems. MALDI-TOF representative spectra acquired in the positive ion mode for single intact bovine oocytes matured in different systems; the intensity presented is relative to the most intense ion pick, and each pick represents one phospholipid. (A) In vitro-maturated oocytes from slaughterhouse ovaries (CONT; n = 12); (B) in vitro-maturated oocytes from non-stimulated animals (IMA; n = 13); (C) in vitro-maturated oocytes from FSH stimulated animals (FSH; n = 13) and (D) in vivo-maturated oocytes (MII; n = 10).

Following the analysis of principal components (PCA), data from the phospholipid profile of each oocyte were plotted in a 3D PCA graph, which was able to explain 73% of the variations (Fig 2). The PCA analysis identified clusters of some ions with, 760.6 and 782.6 *m/z* being the most dispersed (P<0.05). However, based on the ANOVA (Table 6), only the cluster of the 760.6 *m/z* corresponding ions was significantly more abundant (P<0.05) in oocytes from the FSH group compared to oocytes from the MII group (Fig 3). According to previously published data [33], the ion 760.6 intensity cluster corresponds to phosphatidylcholine [PC (34:1) + H] ^+^.

**Table 6:** Comparison of the relative intensity of most dispersed ions after mass spectrometry (MALDI-TOF) analyses, of oocytes from different maturation systems. The data are expressed as arbitrary unit intensity and standard deviation (±SD), and each ion represents a different phospholipid.

	760.6		782.6	
Groups	Intensity (± SD)		Intensity (± SD)	
CONT	40155	(±23241)^a^ ^b^	18545	(±11399)
IMA	35642	(±15855) ^a^ ^b^	11860	(±4852)
FSH	47491	(±36301)^a^	14613	(±6774)
MII	13453	(±3326)^b^	11757	(±6748)

The 760.6 ion corresponds to [PC (34:1) + H] ^+^ and 782.6 to [PC (34:6) + H] ^+^ or [PC (34:1) + Na], both phosphatidylcholines (PC).

^ab^ Different letters in the same column indicates statically differences among the treatments after ANOVA according to Tukey’s test (P<0.05).

CONT = oocytes from slaughterhouse ovaries and matured in vitro; IMA = OPU oocytes from non-stimulated animals and matured in vitro; FSH = OPU oocytes from FSH simulated animal and matured in vitro; MII = OPU oocytes after in vivo maturation.

## Discussion

When the results obtained from cryotop vitrification of bovine and human oocytes [1] are compared, a large discrepancy in the survival rates and viability between these two species is observed [2, 12]. One of the factors that could be responsible for the high cryoresistance of human oocytes to the vitrification process is the difference in maturation systems. In humans, ovaries are superstimulated, and the recovered oocytes are matured in vivo [12, 34]. In contrast in cattle, oocyte maturation usually occurs in vitro, and the females do not receive any superstimulatory treatment [35].

Based on those observations, we hypothesized that in vivo-matured oocytes, which are presumed to be better quality than those matured in vitro, would be more resistant to vitrification and would increase the outcome of in vitro embryo production post-warming.

Because the majority of bovine in vivo maturation systems that have been reported in the literature were developed for *Bos taurus* [25, 36, 37], we wanted to confirm that those protocols were also capable of produce metaphase II (MII) oocytes in *Bos indicus* animals prior to initiating the experiment. A preliminary experiment was conducted in which OPU was performed 20 or 24 h following the injection of a GnRH analog. Based on the results of that pre-experiment, we set the ideal time point to recover in vivo maturated oocytes at 24 hours following GnRH administration.

Regardless of the treatment used, the number of follicles present in the ovaries at the day of OPU was approximately 20, which is in agreement with published observations in *Bos indicus* breeds [38]. The difference between animals that were or were not superstimulated with FSH was evident when considering the sizes of the visible follicles. Superstimulated animals presented a greater percentage of follicles larger than 6 mm in diameter compared to non-superstimulated animals; however, the follicle number was similar between the two groups. This finding is in disagreement with data reported by Blondin *et al*. [39], where Holstein females showed increased follicular diameter and number following FSH stimulation. These differences in follicular population are possibly due to subspecies particularities, as our study was conducted using Nellore animals (*Bos Taurus indicus*) that have a higher follicular population compared to *Bos Taurus* breeds [40].

The concept that higher blood vascularization in the follicle indicates ovulation proximity [26] was demonstrated when we scanned the ovaries following all treatments using color Doppler just prior to OPU. The majority of the follicles present in animals that received GnRH (MII group) showed intense blood vascularization, while in the superstimulated-only (FSH) group, most of the follicles showed moderate vascularization. In the non-stimulated animals (IMA group), the follicle blood vascularization was absent. This information can be used as a tool to determine the follicles that are closest to ovulation and to ensure that the matured oocytes can be recovered when larger follicles with intense blood vascularization are aspirated.

Unexpectedly, the percentage of viable oocytes in the MII group was similar to what was observed for the CONT group, which was not superstimulated. However, it should be noted that in the MII group, only oocytes that were considered to be in vivo-matured were classified as viable. The most accurate method to classify an oocyte as being mature is the visualization of the first polar body. However, in bovine oocytes, this would require the complete removal of the cumulus cells [36], which requires more handling time and may affect oocyte viability [41]. Therefore, to prevent any damage to the oocytes, we chose not to remove the cumulus cells, and to ensure that we were only using healthy, in vivo-matured oocytes, we only considered those showing expanded cumulus cells and/or a visible first polar body to be viable. Thus, some healthy oocytes may have been excluded.

Next, we evaluated the embryonic development of oocytes that had been matured in the different systems and were either fresh or vitrified/warmed. To control for our embryo production system, another group was included consisting of oocytes obtained from slaughterhouse ovaries that had been matured in vitro. Corroborating the results described in the literature, we observed that the blastocyst rates were higher for oocytes obtained from superstimulated heifers [25, 39] compared to those from unstimulated females or from slaughterhouse ovaries. Embryos from the CONT group had lower numbers of cells than the embryos from the MII group. These observations are likely to be related to the greater heterogeneity of slaughtered animals, where nutrition and age could not be controlled. Indeed, the hatching rate on D8, a quality parameter, was lower in blastocysts produced from slaughterhouse ovaries than in those obtained from OPU, reflecting the effect of the oocyte on the final quality of the embryos.

Blastocyst development and hatching rates were notably higher in oocytes that had been exposed to FSH (FSH and MII) compared those that were not exposed (CONT and IMA). According to Rizos *et al*. [25], Dieleman *et al*. [22] and van de Leemput *et al*. [23], in vivo maturation is the gold standard for oocyte maturation and is the ideal system to produce oocytes of higher quality compared to in vitro maturation systems. Despite these studies, we did not find differences in embryo development, hatching rate percentage or total cell number at D8 from in vivo-matured oocytes (MII group) compared to in vitro-matured oocytes retrieved from superstimulated heifers (FSH group). Regarding the immature oocytes that must be matured in vitro, Blondin *et al*. [39] demonstrated that superstimulation with FSH may increase oocyte quality, resulting in improved embryo development. It has been proposed that during the dominance phase of an unstimulated ovary, the dominant follicle will suppress the acquisition of developmental competence by its own COC and by those from subordinate follicles [42]. The loss of dominance signals the COC and might increase their intrinsic developmental competence. Our work is the first study to use *Bos indicus* animals to compare in the same experiment in vivo maturation systems in both immature oocytes and immature oocytes retrieved from stimulated animals. Our results suggest that the administration of GnRH induces a LH surge, and this pre-ovulatory- type follicular environment is necessary to trigger COCs to complete their cytoplasmic maturation, resulting in developmentally competent oocytes [39, 43].

Although we expected that in vivo-matured oocytes would show better embryo development than those that were matured in vitro, the results did not affect our study. The difference in oocyte quality was very clear between the groups that were exposed (FSH and MII) and not exposed (IMA and CONT)to FSH, and these two populations were essential to test our main hypothesis that better-quality oocytes would be more resistant to vitrification/warming. Our results showed that improving oocyte quality was not enough to increase their resistance to cryopreservation. Embryo development from vitrified bovine oocytes did not exceed 5%, even for those derived from in vivo conditions; however, these results are in agreement with other reports [2, 8, 14]. The best results were published by Zhou *et al*. [2], with 10% blastocyst development after oocyte vitrification. Our group had previously observed developmental rates similar to those observed in the present study [14]. Despite both studies having been carried using Cryotop methodology, this discrepancy might be because different sub-species of animals were used.

The oocyte is a cell with peculiarities in areas such as triglyceride storage and phospholipid composition that may play important roles in their resistance to vitrification/warming [16, 44, 45]. Due to the importance of plasma membrane fluidity for resistance to cryopreservation, Zeron *et al*. [46], Horvath *et al*.[4] and our group [14] have investigated methodologies to change the plasma membrane composition. Sudano *et al*. [21] demonstrated that the membrane phospholipid composition can be modified depending on the composition of the culture media or whether the embryo is produced in vivo or in vitro. Due to these findings, we aimed to determine whether the maturation system affects the membrane phospholipid composition of bovine oocytes by MALDI-TOF analysis. Despite the occasional difference observed in the phosphatidylcholine [PC (34:1) + H]^+^ ratio among MII and FSH oocytes, the maturation system did not change the phospholipid profile of Nellore oocytes. The ion we found to be different between in vivo- and in vitro-matured oocytes obtained from superstimulated females has been shown to be affected by subspecies and origin of the embryos [21]. It is less-abundant in Nellore than in Simmental and less-abundant in vivo- vs. in vitro-produced embryos. This PL has a large carbon chain and just one instauration, these two characteristic would result in less cell permeability during cryopreservation process [33] However, we could not correlate the changes detected in oocytes with their origin nor with their ability to develop into embryos after cryopreservation. Therefore, it is possible that either the change was insufficient to alter membrane fluidity or the change in the plasma membrane was not responsible for the damage that occurs during vitrification and warming.

In summary, our results suggest that despite the fact that in vivo maturation systems are capable of producing a highly competent oocyte and produced a change in the plasma membrane lipid when animals are superstimulated, they do not increase embryo development following oocyte vitrification and warming.
